# Regulation of chondrocyte apoptosis in osteoarthritis by endoplasmic reticulum stress

**DOI:** 10.1016/j.cstres.2024.11.001

**Published:** 2024-11-06

**Authors:** Renzhong Li, Kui Sun

**Affiliations:** 1 Taizhou Hospital of Traditional Chinese Medicine, Taizhou, Jiangsu Province, China; 2 The Second Affiliated Hospital of Anhui University of Traditional Chinese Medicine, Hefei, Anhui Province, China; 3 Anhui Acupuncture Hospital, Hefei, Anhui Province, China

**Keywords:** Osteoarthritis, Endoplasmic reticulum stress, Apoptosis, Unfolded protein response, Review

## Abstract

Osteoarthritis (OA), a common degenerative joint disease, is characterized by the apoptosis of chondrocytes as a primary pathophysiological change, with endoplasmic reticulum stress (ERS) playing a crucial role. It has been demonstrated that an imbalance in endoplasmic reticulum (ER) homeostasis can lead to ERS, activating three cellular adaptive response pathways through the unfolded protein response to restore ER homeostasis. Mild ERS exerts a protective effect on cells, while prolonged ERS that disrupts the self-regulatory balance of the ER activates apoptotic signaling pathways, leading to chondrocyte apoptosis and hastening OA progression. Hence, controlling the ERS signaling pathway and its apoptotic factors has become a critical focus for preventing and treating OA. This review aims to elucidate the key mechanisms of ERS pathway-induced apoptosis, associated targets, and regulatory pathways, offering valuable insights to enhance the mechanistic understanding of OA. It also reviews the mechanisms studied for ERS-related drugs or compounds for the treatment of OA.

## Introduction

Osteoarthritis (OA) is a complex joint disorder involving various joint tissues, such as cartilage, meniscus, synovium, infrapatellar fat pad, and subchondral bone. It is caused by a combination of systemic vulnerability and local factors.^1^ The pathogenesis of OA is influenced by several factors, including mechanical stresses, inflammation, aging, and metabolic changes. Characteristic OA symptoms encompass pain, swelling, and stiffness, predominantly in weight-bearing joints, especially the knee and hips. These symptoms eventually lead to joint structure destruction and synovial joint dysfunction, significantly impacting patients’ daily lives.^4^, ^2^, ^3^ In China, the prevalence of OA ranges from 0.34% to 0.36%, with approximately half of individuals over 50 years old being susceptible to OA.^5^ OA is characterized by the progressive deterioration of articular cartilage, metabolic disruptions in the usual cartilage matrix, subchondral bone sclerosis, and the development of subchondral cysts and bone densities.^6^ Despite considerable research efforts, the pathogenesis of OA remains incompletely elucidated. OA development is influenced by several factors, including the release of inflammatory factors, mechanical injury to articular cartilage, and the aging of chondrocytes, all of which contribute significantly to the considerable damage seen in articular cartilage.^7^ Currently, no pharmacologic interventions effectively halt disease progression or mitigate cartilage damage.^8^ The challenge in treating OA is intricately linked to the unique characteristics of joint tissues, particularly articular cartilage.^9^ Cartilage comprises an extracellular matrix and chondrocytes, which oversee extracellular matrix biogenesis and maintenance. Over time, chondrocytes accumulate damage caused by mechanical overload, DNA damage, inflammatory factor release, and both mechanical and metabolic imbalances.^11^, ^10^ These factors collectively contribute to an upsurge in the production of reactive oxygen species, initiating oxidative stress (OS) within chondrocytes.^12^ OS assumes a pivotal role in the development of OA. Excessive OS disrupts calcium ion homeostasis in the endoplasmic reticulum (ER), triggering endoplasmic reticulum stress (ERS). When ERS is elevated or prolonged, it culminates in chondrocyte apoptosis, extracellular matrix degradation, heightened systemic inflammation, and articular cartilage degeneration, ultimately advancing OA. In OA, articular cartilage experiences heightened cellularity, with reduced chondrocyte proliferation and an increased incidence of apoptosis.

The ER is crucial for folding and processing polypeptide chains into functional proteins within cells. Both internal and external factors can cause the buildup of misfolded or unfolded proteins in the ER, resulting in ER stress, or ERS.^13^, ^14^ Factors like hypoxia, OS, and nutritional deficiencies can activate pathways in the ER related to cell death, ultimately leading to apoptosis.^15^, ^16^ ERS represents a critical cellular defense mechanism aimed at enhancing resistance to both internal and external stressors. In multicellular organisms, sustained or serve ERS can instigate programmed cell death, specifically apoptosis.^17^ When cartilage sustains damage, newly synthesized proteins are secreted to facilitate the repair of the compromised extracellular matrix. However, this process disrupts the internal equilibrium of cartilage, leading to chondrocyte apoptosis.^18^ Chondrocytes, the main cellular element in articular cartilage, are crucial for cartilage metabolism.^19^ The loss of chondrocytes through apoptosis, referred to as chondrocytopenia, is a key pathological characteristic of OA. Importantly, chondrocyte apoptosis is a significant factor in both the onset and advancement of OA, with ERS playing a vital role in its control. The effective mitigation of ERS holds promise as a means to reduce chondrocyte apoptosis, consequently mitigating cartilage loss in individuals with OA. This approach has the potential to prevent and manage both the onset and progression of OA.

This review outlines the current understanding of signaling pathways associated with the unfolded protein response (UPR) during ERS. It discusses potential causes of dysfunctional stress processes in chondrocytes and emphasizes ERS-induced apoptosis in articular chondrocytes during OA. Targeting endoplasmic reticulum stress (ERS) or modulating UPR signaling to decrease chondrocyte apoptosis holds promise for future OA therapies.

## Overview of ERS

ERS refers to a phenomenon wherein various pathophysiological factors impact an organism, resulting in reduced intracellular nutrients and imbalances in Ca^2+^ levels. This, in turn, impairs protein processing, the transport capacity of the ER, and disrupts Ca^2+^ uptake and release, ultimately hindering the normal physiological function of ER.^20^ ERS primarily activates three signaling pathways: the UPR, the ER-overload response, and the cholesterol regulatory cascade effect.^21^ Among these, the UPR pathway is central to both protective and apoptotic signaling during ERS and has been more extensively studied.^22^ The UPR is a cellular mechanism that regulates ER protein homeostasis and is normally inactive. Three transmembrane-sensing proteins within the UPR pathway—pancreatic endoplasmic reticulum kinase (PERK), inositol-requiring enzyme 1 (IRE1), and activating transcription factor 6 (ATF6)—are bound and inactivated by glucose-regulated protein 78 (GRP78).^23^ However, conditions such as gene mutations, hypoxia, nutrient deficiencies, OS, and the accumulation of unfolded proteins can induce the UPR. In response, the three transmembrane proteins dissociate from GRP78 and activate it, thus alleviating early ERS.^24^, ^25^ When cells experience ERS, the activated UPR can lead to two contrasting outcomes. On one hand, if the UPR effectively reduces the amount of unfolded proteins, it becomes inactive, and ER homeostasis is restored to a physiological state, enabling cell survival. On the other hand, if the UPR fails, severe ERS ensues, resulting in cell death or apoptosis through multiple pathways.^26^ The UPR aims to address the accumulation of unfolded proteins through several mechanisms. The UPR downregulates the transcription of secreted proteins and promotes the elimination of misfolded or slowly folding proteins through endoplasmic reticulum-associated degradation or lysosomal degradation.^27^ Additionally, it increases the synthesis of ER resident chaperones and folding enzymes, enhancing ER folding capacity and reducing the burden of unfolded proteins.^28^, ^29^ The UPR predominantly relies on three prosurvival signaling pathways: PERK–eIF2α, IRE1–X box-binding protein 1 (IRE1–XBP1), and ATF6. These pathways elevate the expression of ERS-associated proteins, inhibit the transcription of Message RNAs (mRNAs) that enhances ER folding ability, and facilitate the degradation of unfolded proteins to adapt to ERS.^30^ Under conditions of ERS, the UPR pathway can either promote cell survival or induce apoptosis.

## ERS-mediated apoptosis signaling pathway

Apoptosis, a programmed cell death mechanism, is characterized by DNA degradation, nuclear concentration, fragmentation, and the generation of apoptotic vesicles with intact cell membranes, which do not induce secondary inflammatory reactions. This process, essential for life maintenance, primarily occurs through two pathways: the extracellular death ligand-receptor pathway and the intracellular pathway. Apart from the well-known death receptor and mitochondrial pathways,^31^ significant focus has been placed on the ERS-initiated apoptosis pathway.

When cellular damage exceeds the UPR's ability to cope with the increased load of unfolded proteins and normal ER function cannot be restored, the UPR shifts from prosurvival to proapoptotic signals. This is characterized by the activation of the three transmembrane signaling pathways of the UPR, which then activate downstream apoptotic signaling markers such as C/EBP homologous protein (CHOP)/GADD153, caspase-12, Jun N-terminal kinase (JNK), and other signaling molecules, initiating apoptotic responses.^32^ Within the UPR, PERK, IRE1, and ATF6 have been identified as inducers of apoptosis in the ERS state.^33^, ^34^ While the UPR diligently strives to repair damaged cells, it also selectively eliminates those beyond salvage to prevent further harm to the organism.^35^

### Transcriptional activation of CHOP/GADD153 gene

CHOP/GADD153 serves as an ERS-specific transcription factor, maintaining low expression levels under normal conditions. Nevertheless, in ERS-induced apoptosis, the activation of IRE1, PERK, and ATF6 results in a significant rise in CHOP expression, ultimately leading to apoptosis. Increased CHOP levels are linked to decreased expression of B-cell lymphoma-2 (Bcl-2), an antiapoptotic protein, thereby facilitating apoptosis.^36^, ^37^

### JNK activation pathway

In ERS response, IRE1 activation can lead to the assembly of JNK and tumor necrosis factor receptor-associated factor 2 (TRAF2). This complex formation then activates apoptosis signal-regulating kinase 1 (ASK1), leading to the formation of a trimer comprising IRE1, TRAF2, and ASK1. This trimeric complex initiates apoptosis *via* the JNK pathway.^39^, ^38^

### Caspase-12 activation

Caspase are a family of aspartate-specific cysteine protein hydrolases, with caspase-3 being a prominent protease in the apoptotic execution phase, situated at the downstream end of the caspase cascade. Specifically associated with ER stress-induced apoptosis is caspase-12, which is activated exclusively during ERS and not within the mitochondrial or death receptor apoptotic pathways.^40^ When ERS occurs, caspase-12 can be activated by the formation of a complex between TRAF2 and IRE1.^41^ Subsequently, calpain activation, mediated by the release of Ca^2+^ into the cytoplasm through the CHOP–ERO1α (ER oxidase 1α) pathway, cleaves and activates caspase-12. This activation, in turn, triggers other caspase effector proteins, ultimately resulting in apoptosis.^42^ Caspase-7 also relocates to the ER surface, forming a complex with caspase-12, further enhancing caspase-12 activation and stimulating other caspase effector proteins, thus facilitating apoptosis.^43^

## ERS induces chondrocyte apoptosis in OA

In the onset of OA, alterations in the original pressure and oxygen gradient within articular cartilage stimulate chondrocytes into a state of stress. This disruption in homeostasis subsequently leads to protein misfolding and ER accumulation, prolonged ERS response.^44^ This prolonged ERS response disrupts the signaling cascade, thereby triggering ERS-induced apoptosis.^45^ It has been found that the duration and severity of OA correlate with the extent of chondrocyte apoptosis, emphasizing the critical role of chondrocyte apoptosis in OA progression.^46^, ^47^ Studies by Oliver *et al.*^48^ and Tsang *et al.*^49^ have also demonstrated the presence of ERS in chondrocytes. Given that chondrocytes can sense changes in the intra-articular microenvironment and regulate the metabolic balance of the cartilage matrix, their survival status and influence on cartilage matrix metabolism directly impact the onset and progression of joint diseases.^50^ Takada *et al.*^51^ have proposed that inhibiting ERS-induced chondrocyte apoptosis could provide a novel and effective avenue for preventing and treating OA.

**Fig. 1:**
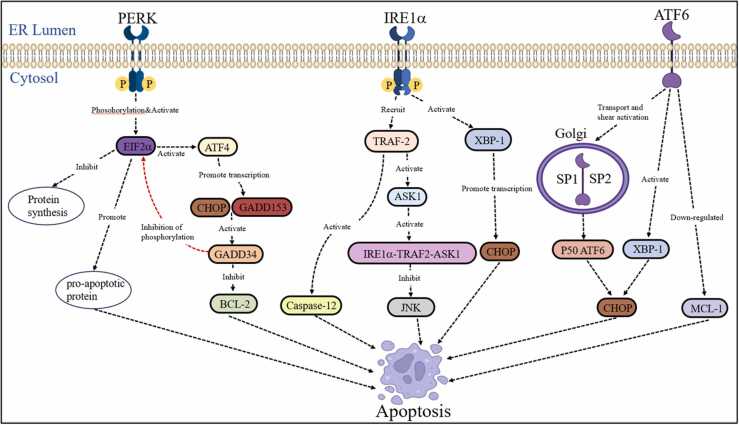
Signaling pathways of endoplasmic reticulum stress-mediated chondrocyte apoptosis in osteoarthritis. Mediated by three major pathways, PERK, IRE1α, and ATF6, all three transmembrane sensors induce apoptosis. Abbreviations used: ASK1, apoptosis signal-regulating kinase 1; ATF4, activating transcription factor 4; ATF6, activating transcription factor 6; CHOP, C/EBP homologous protein; EIF2α, eukaryotic initiation factor 2α; ER, endoplasmic reticulum; IRE1, inositol-requiring enzyme 1; JNK, Jun N-terminal kinase; PERK, pancreatic endoplasmic reticulum kinase; TRAF2, tumor necrosis factor receptor-associated factor 2; XBP1, X box-binding protein 1.

Persistent and severe ERS in chondrocytes impair ER function, triggering the immediate activation of the apoptotic pathway through IRE1, PERK, and ATF6 signaling. This activation subsequently involves downstream apoptotic signaling markers, including CHOP/GADD153, JNK, and Caspase-12, ultimately mediating chondrocyte apoptosis (Figure 1).

### IRE1 signaling pathway and induced chondrocyte apoptosis

IRE1 is a transmembrane protein featuring two functional domains: an N-terminal luminal sensor domain and a C-terminal cytosolic effector domain. There exist two isoforms of IRE1: IRE1α and IRE1β.^52^ IRE1α is a single transmembrane protein with distinctive luminal and cytoplasmic structural domains, each carrying out specific functions on opposite sides of the ER membrane.^53^ IRE1α possesses both protein kinase and endonuclease activities. Upon the accumulation of unfolded proteins in the ER, IRE1α undergoes oligomerization and autophosphorylation, thereby activating its ribonuclease activity. When protein misfolding in the ER exceeds the UPR's handling capacity, IRE1α triggers a process called regulated IRE1-dependent decay (RIDD). By splicing XBP1 *via* IRE1α, cells enhance survival; however, under ERS conditions, RIDD promotes apoptosis by degrading messenger RNAs that encode survival factors and micro-RNAs that target proapoptotic factors. As protein misfolding in the ER overwhelms the UPR's processing ability, IRE1α oligomerizes and undergoes autophosphorylation, activating its ribonuclease function.^54^ This activation initiates RIDD, ultimately promoting apoptosis during ERS.^55^ During ERS, cells initiate the UPR to mitigate stress and minimize cellular damage, with IRE1α primarily exhibiting XBP1 splicing activity in this context. Upon activation, IRE1α splices XBP1 mRNA, generating mature and active XBP1 protein. This active form enhances ER protein folding, mitigating cellular damage from ERS.^57^, ^56^ It also binds to gene promoters involved in UPR and endoplasmic reticulum-associated degradation, including CHOP, to restore protein homeostasis. Consequently, XBP1(s) can upregulate CHOP expression.^60^, ^58^, ^59^ IRE1α can activate ASK1, which in turn activates downstream kinases like JNK and p38 MAPK, culminating in apoptosis.^52^ Active JNK regulates Bcl-2 family members, activates proapoptotic factors, and executes apoptosis through caspases.^61^ The p38 MAPK family phosphorylates CHOP at Ser78 and Ser81, further inducing apoptosis.^63^, ^62^ It has been found that, when the IRE1 signaling pathway is overstimulated by ERS, activated IRE1α binds to TRAF2 and ASK1, initiating the JNK pathway. The formation of the IRE1α–TRAF2–ASK1 complex activates JNK, which plays a role in mediating apoptotic cell death during irreversible ERS.^64^ Additionally, Wei *et al.*^65^ found that dense granule protein 15 induces IRE1α phosphorylation, leading to the activation of XBP1 and XBP1s, resulting in increased CHOP expression. Alternatively, GRA15 can recruit TRAF2, which activates ASK1 and downstream JNK, inducing apoptosis in choriocarcinoma JEG-3 cells.

During ERS, the molecular chaperone Bip dissociates from the tubulo-structural domain of IRE1α. In a study using a mouse model of trauma-induced medial meniscus dislocation, a notable increase in Bip expression was observed, suggesting a role for ERS and UPR in OA pathogenesis.^66^ In OA chondrocytes, the JNK signaling pathway activates Bcl-2, contributing to chondrocyte apoptosis during cartilage degeneration.^67^ Elevated levels of active JNK and active p38 MAPK were detected in human OA cartilage compared to normal cartilage, suggesting a link between this pathway and cell death in articular chondrocytes.^68^ A recent study also noted increased expression of the proapoptotic protein Bax in OA chondrocytes, highlighting the crucial role of the IRE1α–TRAF2–ASK1 pathway in chondrocyte apoptosis.^69^

The mechanism of cyclic RNA in chondrocyte apoptosis has gained significant attention in current research. In an interleukin-1β-induced chondrocyte injury model, cyclic RNA-0114876 was found to increase the expression of TRAF2. Conversely, inhibiting cyclic RNA-0114876 enhanced chondrocyte activity, reduced inflammatory responses, and mitigated apoptosis. These findings suggest that cyclic RNAs may play a significant role in chondrocyte apoptosis through the IRE1α–TRAF2–ASK1 signaling pathway.^70^ Recent discoveries have also highlighted decreased levels of miR-502-5p in OA joint tissues and IL-1β-induced chondrocytes. MiR-502-5p was found to protect against chondrocyte apoptosis by targeting TRAF2, indirectly suggesting its involvement in the IRE1α–TRAF2–ASK1–JNK signaling pathway through ERS.^71^

### PERK signaling pathway and induced chondrocyte apoptosis

PERK, a transmembrane protein, plays a crucial role as a sensor in the UPR by regulating OS and dampening protein translation. When unfolded proteins accumulate in the ER, PERK undergoes oligomerization and autophosphorylation, leading to the phosphorylation of eukaryotic initiation factor 2α (eIF2α).^72^ Phosphorylated eIF2α further enhances the activation of activating transcription factor 4 (ATF4), a stress-responsive transcription factor that regulates genes involved in redox homeostasis, amino acid metabolism, protein synthesis, apoptosis, and autophagy. During ERS, ATF4 accumulates at target gene promoters, dephosphorylates eIF2α, and regulates the expression of CHOP, DNA damage-inducible protein 34 (GADD34), and activating transcription factor 3 (ATF3) by upregulating protein phosphatase 1. This process helps restore protein synthesis following ERS.^73^, ^74^ CHOP, a transcription factor downstream of ATF4, is produced significantly following prolonged ERS. It promotes macrophage apoptosis and ERS-induced cytokine production.^75^ Although PERK and ATF4, along with eIF2α S51A knock-in cells, are unable to induce CHOP during ERS, sustained intense ERS activates downstream apoptotic signaling, initiating the PERK–ATF4–CHOP apoptotic pathway.^76^ However, previous studies suggest that CHOP alone may not suffice to induce cell death, emphasizing the necessity for cooperation between CHOP and ATF4 to induce apoptosis.^77^, ^78^ CHOP contributes to eIF2α dephosphorylation by binding to the AARE1 element of the CHOP promoter, forming a heterodimer with ATF4, and upregulating transcription of growth inhibitory and DNA-damage GADD34. This, in turn, results in increased protein synthesis and apoptosis.^79^ Research has shown a significant upregulation of the proapoptotic gene CHOP/GADD153 in cases of severe ERS.^80^ In OA, the loss of structural collagen type II inhibits the natural increase of type I collagen within the cartilage. This disruption to the integrity of the extracellular cartilage matrix has a significant impact on cartilage properties. Data demonstrate that decreased PERK expression in OA chondrocytes results in reduced expression of type II collagen and increased expression of type I collagen.^81^ These alterations contribute to microenvironmental shifts in the extracellular matrix, ultimately fostering cartilage degeneration and promoting the degenerative process.^81^ Consequently, boosting PERK levels in OA cartilage holds therapeutic potential, as these microenvironmental shifts directly contribute to cartilage degeneration. Based on these findings, elevating PERK levels may represent a therapeutic avenue for OA cartilage.^82^ The activation and detachment of the specific molecule GRP78 occur when ERS is detected. Subsequently, PERK takes the lead in phosphorylation, catalyzing the phosphorylation of eIF2α, leading to an upregulation of CHOP expression. This imbalance between Bcl-2 and the caspase family activates a cascade of apoptotic signals, including GRP78–PERK–CHOP, and related downstream responses, ultimately culminating in chondrocyte apoptosis.^83^ The PERK-mediated UPR acts protectively against various internal and external stresses by boosting ATF4 production. ATF4, in turn, contributes to CHOP transcription. While all three UPR pathways can induce CHOP transcription, the PERK–eIF2α–ATF4 pathway significantly impacts CHOP production. In a mouse model of OA, articular cartilage degeneration and chondrocyte apoptosis were less severe in mice lacking the CHOP gene compared to normal mice,^84^ indicating that CHOP-mediated UPR worsens OA.

Several studies have indicated that an increase in the expression of ERS-related factors, such as GRP78, was observed in the Lipopolysaccharide (LPS)-induced OA cell model. In contrast, the levels of GRP78, ATF-6, CHOP, p-PERK/PERK, and p-eIF2α/eIF2α were significantly reduced in chondrocytes after treatment with albuterol. This suggests that albuterol may inhibit the activation of the GRP78–PERK–CHOP signaling pathway, consequently hindering apoptotic cell death.^83^, ^85^ signaling pathway activation to inhibit LPS-induced chondrocyte apoptosis. Targeting ERS and interfering with UPR signaling to reduce chondrocyte apoptosis represent effective strategies for the treatment of OA.^85^

### ATF6 signaling pathway and induced chondrocyte apoptosis

ATF6, a type II transmembrane protein found in two isoforms, ATF6α and ATF6β, is widely expressed in mammals. During ERS, ATF6 translocates to the Golgi compartment, where it is activated by S1P and S2P cleavage. This activation generates the active transcription factor fragment ATF6 P50, which regulates the expression of ERS-specific genes.^86^ Studies have shown that Small interfering RNA (siRNA)-mediated silencing of ATF6 initially reduces CHOP induction during the acute phase but prolongs CHOP expression, highlighting ATF6's role as a crucial regulator in determining cell fate under chronic ERS conditions.^87^ Upon activation, ATF6 translocates to the nucleus, where it forms homodimer or heterodimer that interacts with ATF/cAMP response elements and ERS response elements on gene promoters,^74^ including those of CHOP, GRP78, and XBP1. This interaction promotes the transcription of these target genes.^89^, ^88^ While ATF6 was traditionally thought to transmit survival signals during ERS, recent studies have demonstrated that ATF6 overexpression induces CHOP mRNA expression, and ATF6 mutations inhibit ERS-induced CHOP expression. ATF6 can activate both CHOP and XBP1 transcription, with XBP1 further regulating CHOP expression. Consequently, ATF6 can cooperate with XBP1 to activate CHOP.^90^ Additionally, ATF6 was also found to increase apoptosis through the downregulation of the antiapoptotic protein Myeloid Cell Leukemia-1 (MCL-1).^91^ Zhou *et al.*^92^ found that ATF6 plays a crucial role in preventing the progression of chronic pancreatitis by regulating p53-mediated apoptosis, as evidenced by increased apoptosis and p53 expression in a mouse model of chronic pancreatitis during disease progression.

The ATF6 pathway seems to have limited relevance to OA. Although ATF6α is linked to ERS sensing and XBP1 signaling, ATF6α knockout mice have normal articular cartilage and show a similar incidence of OA as wild-type mice following DMM surgery.^66^ It is possible that ATF6β compensates for ATF6α in knockout animals. ATF6β plays a significant role in regulating proliferation and aggregate enzyme expression in growth plate chondrocytes, which could be relevant to OA.^94^, ^93^ Xie *et al.* found that ATF6 is a target gene for miR-31-5p, revealing the mechanism by which miR-31-5p mediates chondrocyte apoptosis and calcification.^95^ Elevated levels of miR-31-5p inhibit ATF6, reducing ERS promotion in chondrocytes, thereby reducing apoptosis and calcification. Zhou *et al.*, through downregulating the expression of ERS markers like GRP78, CHOP, ATF6, and caspase-12 with naphthocorticosteroid or 4-phenylbutyric acid (4-PBA) after Interleukin-1β (IL-1β) treatment, concluded that daphnoretin effectively curtailed chondrocyte apoptosis.^96^ Currently, numerous experimental studies focusing on ERS and OA have been reported.^97^, ^82^, ^85^ It is conceivable that exploring effector molecules that inhibit chondrocyte apoptosis within the ERS-related signaling pathway and employing them as pivotal targets to obstruct ERS and diminish chondrocyte apoptosis could significantly mitigate cartilage loss in OA patients. This approach holds the potential to prevent and manage the progression of OA, offering an effective pathway for its treatment.

## Treatment: Drugs and chemical compounds targeting the ERS-mediated apoptosis pathway

**Fig. 2:**
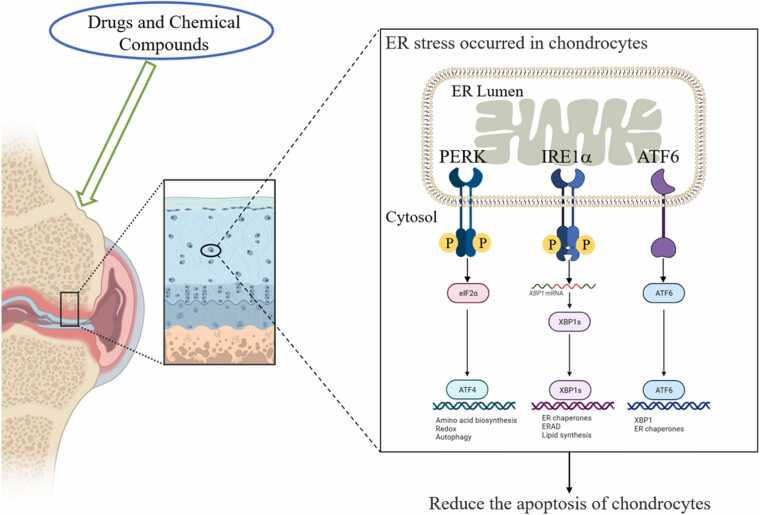
Restoration of ER homeostasis as a therapeutic target in OA. Drugs or compounds aim to restore the balance between prosurvival and proapoptotic ERS to achieve protective outcomes in chondrocytes under stress. The main approaches are as follows: (i) direct alleviation of ERS by reducing protein misfolding through the use of drugs or compounds, (ii) the use of compounds capable of directly augmenting the protective response to UPR, and (iii) the use of a drug or a compound that prevents the triggering of ERS-mediated apoptosis through the inhibition of CHOP, cystathionase 12, and JNK activity and expression. Abbreviations used: ATF4, activating transcription factor 4; ATF6, activating transcription factor 6; EIF2α, eukaryotic initiation factor 2α; ER, endoplasmic reticulum; ERAD, endoplasmic reticulum-associated degradation; IRE1, inositol-requiring enzyme 1; PERK, pancreatic endoplasmic reticulum kinase; XBP1, X box-binding protein 1.

The ERS-mediated apoptotic pathway may be a valuable therapeutic strategy to control ERS associated with OA. Recently, many drugs and compounds capable of apoptosis through ERS of the UPR have been identified and tested. This review will present some of the most widely used drugs as promising therapeutic candidates (Table1 and Figure 2).

**Table 1:** Drugs and compounds induce ERS-mediated chondrocyte apoptosis.

Drugs and chemical compounds	Model	Action mechanism
Brazilin	Rats	Decrease GRP78 and increase CHOP
Chrysin (CHR)	Human chondrocyte	Inhibition of GRP78/PERK/CHOP signaling pathway
Aucubin	Rats	ERS pathway regulates Bcl-2, caspase-9, caspase-3 expression
ABPS	Mice	PERK signaling pathway
curcumin	Rats	PERK–eIF2α–ATF4–CHOP
Echinacoside (ECH)	Rats	PERK–eIF2α–ATF4–CHOP
Salicin (SA)	Rats	IRE1α–IκBα–p65
Vitexin	Rats	Decrease GRP78 and increase CHOP
T-2 toxin	Rats	PERK–eIF2α–ATF4–CHOP
Celastrol	Rats	ATF6–CHOP
Histone deacetylase 4 (HDAC4)	Chondrocyte	p53-Dependent ERS pathway regulates caspase-12 and caspase-3 expression
4-Phenylbutyric acid (4-PBA)	Rats	Regulates apoptosis through the expression of Bip and CHOP

Abbreviations used: ATF6, activating transcription factor 6; CHOP, C/EBP homologous protein; EIF2α, eukaryotic initiation factor 2α; ERS, endoplasmic reticulum stress; GRP78, glucose-regulated protein 78; PERK, pancreatic endoplasmic reticulum kinase.

Brazilin is the main active ingredient of sappan wood. It has been shown that Brazilian can inhibit the release of Tumor necrosis factor-α (TNF-α) and IL-1β to realize the anti-inflammatory function.^98^ TNF-α, IL-1, and Interleukin-6 (IL-6) levels were found to be abnormally elevated in the synovial fluid, synovium, and cartilage of OA patients.^100^, ^99^ Huang *et al.*^97^ obtained that Brazilian reduced the expression level of GRP78 and increased the expression of CHOP in an IL-1β-induced OA cell model and concluded that Brazilian inhibited ERS-induced apoptosis of chondrocytes in an IL-1β-induced OA cell model and effectively reversed the expression of MMP-13 and collagen type II expression, suggesting that Brazilian has a certain repairing effect on OA cartilage damage.

Chrysin (CHR) is a flavonoid with various pharmacological activities, which exists in many plants, such as wood butterfly and *Scutellaria baicalensis*.^103^, ^101^, ^102^ Studies have shown that CHR can alleviate IL-1β-induced human OA by inhibiting nuclear factor-κB (NF-κB) activation and high-mobility group protein B1 signaling pathway. Chondrocyte apoptosis and inhibit inflammation, promoting chondrocyte secretion of type II collagen and maintaining normal cartilage function.^104^ Another study showed that CHR inhibited ERS and oxidative damage in diabetes.^105^ Enhanced expression of ERS-related factors such as GRP78 was observed in the LPS-induced OA cell model, whereas GRP78, ATF6, CHOP, pPERK/PERK, and p-eIF2α/eIF2α expression was significantly reduced in chondrocytes after treatment with salicin (SA), suggesting that SA may inhibit the activation of the GRP78/PERK/CHOP signaling pathway through inhibition of LPS-induced chondrocyte apoptosis.^106^

Aucubin is a natural compound isolated from Cortex Eucommiae. Young *et al.*^107^ reported that aucubin could affect caspase-3 activity and apoptosis through ERS and was protective and mechanically stimulatory in a chondrocyte model of H-induced OA. The results of the study by Wang *et al.*^108^ further indicated that aucubin exerts antiapoptotic effects on chondrocytes through ERS regulation of these key mediators (including Bcl-2, Bax, caspase-9, and caspase-3).

Achyranthes bidentata (AB) is a Chinese herbal medicine extracted from AB, which is widely used in the clinical treatment of orthopedic diseases, including OA.^109^, ^110^ Achyranthes bidentata polyscharides (ABPS) are extracted from AB. A previous study confirmed that ABPS could promote the G1/S cell cycle transition, inducing chondrocyte proliferation and inhibiting apoptosis.^111^ ABPS may facilitate the restoration of articular cartilage in mice with OA. Further protein blotting experiments revealed that ABPS inhibited the expression of ERS and inflammation-related factors, such as PERK, Bip, ATF4, CHOP, IL-6, TNF-α, and RELA, whereas ABPS promoted the repair of articular cartilage in OA mice. Further protein blotting experiments revealed that ABPS inhibited the expression of ERS and inflammation-related factors, such as PERK, BiP, ATF4, CHOP, IL-6, TNF-α, and RELA. ABPS intervention reduced the level of lncRNA NEAT1, leading to increased expression of miR-377-3p, and competitively binds to PERK through the PERK signaling pathway to regulate chondrocyte ERS. It has been demonstrated that ABPS can inhibit ERS in chondrocytes by regulating the PERK signaling pathway.^112^

Curcumin, a polyphenolic compound extracted from turmeri.^113^ *In vitro* and *in vivo* studies have demonstrated that curcumin can alleviate apoptosis in both human chondrocytes and animals.^114^, ^115^ Curcumin has been found to attenuate ERS by inhibiting the PERK–eIF2α–ATF4–CHOP pathway. Its study demonstrated, for the first time, that curcumin inhibits ERS-induced apoptosis of chondrocytes *in vitro* and *in vivo*, thereby ameliorating the progression of OA. Curcumin-stimulated inhibition of ERS and the associated PERK–eIF2α–ATF4–CHOP signaling pathway protected rat chondrocytes from apoptosis by promoting SIRT1 expression.^116^

Echinacoside (ECH) is a natural phenylethanol that can be extracted from Cistanche deserticola Ma. Lin *et al.*,^117^ by establishing an OA model in chondrocytes *in vitro*, found that ECH significantly reduced the levels of the ERS marker proteins GRP78, ATF4, and CHOP, as well as phosphorylated PERK and eIF2α. ECH also decreased the level of proapoptotic protein Bax and increased the expression of antiapoptotic protein Bcl-2 to prevent chondrocyte apoptosis. ECH was demonstrated to inhibit the PERK–eIF2α–ATF4–CHOP pathway, thereby attenuating ERS and ultimately reducing apoptosis.

SA, a small molecule extracted from the bark of willow. By *in vitro* and *in vivo* assays, SA alleviated OA by directly binding to the ER stress regulator IRE1α and inhibited chondrocyte apoptosis by suppressing IRE1α-mediated ERS *via* IRE1α-IκBα-p65 signaling.^118^

Vitexin, an active ingredient in hawthorn leaf extracts, was shown to exert protective effects on chondrocytes, by inhibiting the expression of GRP78 and PDI, and an apoptotic protein (CHOP) induced by interleukin-1β. It also modulated thapsigargin-induced upregulation of GRP78 and PDI and subsequently an apoptotic protein (CHOP). Among rat chondrocytes, both the ER stress-activated nuclear factor kappa B pathway and the induced expression of inflammatory cytokines (IL-6 and TNF-α) were significantly inhibited by vitexin. Finally, vitexin attenuated the progression of OA *in vivo* in rats.^119^

T-2 toxin is one of the most cytotoxic type A trichothecene mycotoxins produced by the *Fusarium* fungus. Chondrocytes are extremely sensitive to T-2 toxin, which can cause damage at a dose of 5 ng/mL *in vitro* study,^120^ and *in vivo* study, increased chondrocyte apoptosis occurs in rat knee articular cartilage at a dose of 200 ng/g BW/d.^121^ These evidences have strongly proved that T-2 toxin is an important factor in chondrocyte damage. Liu *et al.*^122^ found that the expression of GRP78, CHOP, and caspase-12 was higher *in vivo* and *in vitro* in the T-2 toxin group than in the control group, and that T-2 toxin promotes apoptosis of chondrocytes, inhibits matrix synthesis, and accelerates cellular catabolism through the ERS signaling pathway. In addition, salubrinal was found to inhibit ERS-mediated apoptosis through the PERK–eIF2α–ATF4–CHOP signaling pathway, thus preventing chondrocyte damage.

Celastrol is a bioactive compound obtained from *Tripterygium wilfordii*.^123^ Celastrol has been demonstrated to exert antiapoptotic effects through its influence on the Akt and p38 MAPK signaling pathways.^124^ Celastrol can attenuate pain and cartilage damage in OA rats. Liu *et al.*^125^ employed clindamycin to develop an *in vitro* OA model. Their findings revealed that celastrol diminished enzyme activity and protein expression of caspase-3, caspase-6, and caspase-9, while also reducing the expression of Bip, ATF6, CHOP, and XBP1 proteins and mRNA levels. Celastrol was observed to impede the progression of OA by inhibiting ER-mediated apoptosis through the ATF6/CHOP signaling pathway. Celastrol has been demonstrated to prevent OA by inhibiting ER-mediated apoptosis through the ATF6/CHOP signaling pathway.

Histone deacetylase 4 (HDAC4) is a class II histone deacetylase that is mainly distributed in brain, muscle, and cartilage.^126^ Studies have shown that decreased HDAC4 expression in cartilage accelerates the pathogenesis of OA, and upregulating HDAC4 expression might become a new treatment method for OA.^128^, ^127^ Cells were treated with a p53 inhibitor by Guo *et al*. It was found that the 1-669aa-induced expression of caspase-12 and caspase-3 could be significantly suppressed by inhibiting the expression of p53, which revealed that 1-669aa may induce chondrocyte apoptosis through the p53-dependent ERS pathway.

4-PBA is a small molecule chemical chaperone that effectively diffuses into cartilage explant cultures and dose-dependently reduces excessive ER stress in chondrocytes.^129^ In rats model of OA caused by cruciate ligament rupture, 4-PBA administered by gavage showed reduced tissue damage, downregulation of ERS markers such as Bip and CHOP, and reduced apoptosis.^130^

## Conclusion and future perspectives

In this review, we summarize the direct pathways of ERS-regulated chondrocyte apoptosis. It should be noted that ERS also regulates apoptosis through the process of cellular autophagy. ERS exerts its regulatory influence over autophagy in two distinct ways: directly through proteins associated with the UPR pathway and indirectly by modulating the concentration of cytoplasmic Ca²⁺. Autophagic cell death represents a distinct regulatory mechanism of programmed cell death, differing from apoptosis. Additionally, autophagy exerts a negative feedback mechanism to inhibit ERS, thereby maintaining the stress threshold for initiating apoptosis and promoting cell survival.^131^ In the event that the intensity of stress surpasses the scope of autophagy regulation or if autophagy functionality is compromised, the apoptotic pathway is initiated, with autophagy-related proteins assuming a proapoptotic role at this juncture. Autophagy is thought to be in balance with apoptosis when there is increased chondrocyte apoptosis occurring with lower expression of autophagy regulators in OA joints of human beings and rodents.^132^ Autophagy seems to be a protective mechanism for stabilized microenvironment in articular cartilage, and less autophagic activity is observed in aging or osteoarthritic joints.^133^ The specific nodes and mechanisms of ERS that indirectly regulate apoptosis through autophagy remain to be elucidated, and there is a paucity of related studies that can be conducted.

It has been identified that ERS-induced apoptosis of chondrocytes contributes to the degeneration of cartilage in OA. OA is a multifaceted condition, and its nonsurgical therapeutic options encompass physical therapy, exercise therapy, weight management, and medication. These interventions primarily aim at symptom management and pain reduction, but their efficacy remains limited.^134^ Knee arthroplasty represents the last resort, despite its associated challenges like surgical complexity and prosthesis positioning intricacies.^135^ Currently, effective strategies for intervening in OA development or halting its progression remain scarce. Extensive research has focused on chondrocytes, aiming to address OA through cartilage repair mechanisms. Notably, ERS-induced chondrocyte demise has emerged as a contributing factor to cartilage degeneration in OA. Consequently, signaling pathways governing ERS provide promising targets for future OA therapeutic approaches.

ERS presents a multifaceted process encompassing intricate substance exchange, organelles signaling, and intricate regulatory interactions among diverse tissue cells, intertwining with apoptosis. The mechanisms of action of IRE1, PERK, and ATF6 have been established. The PERK-related pathway has a lot of experimental content and a more complete research mechanism, which may be of great help to the occurrence and development of OA as well as the research and development of targeted drugs, but it is undeniable that the other two signaling pathways may also have a great influence on OA, and more follow-up studies are needed. The mechanism governing its actions and precise regulation remain subjects demanding further comprehensive exploration. Further investigation is needed to understand the mechanisms and precise regulation of apoptosis, which could lead to potential drug targets for protecting articular cartilage in OA. Recent progress in understanding apoptosis mechanisms has revitalized interest in this area. The interaction between autophagy, ERS, and apoptosis, along with the emerging role of miRNAs in apoptosis regulation, will provide new insights for therapeutic interventions. However, it is not yet fully understood whether apoptosis can be effectively and safely modulated to prevent or reduce chondrocyte apoptosis in OA. A deeper understanding of how these signaling pathways interact with molecular chaperones is crucial.

## Ethics statement

Not applicable.

## Funding and support

This study was supported by 2024 Special Funds for Inheritance and Development of Traditional Chinese Medicine from the Central Government - National TCM Advantageous and Characteristic Specialty Construction Project - Department of Orthopaedics and Traumatology of the Second Affiliated Hospital of Anhui University of Traditional Chinese Medicine (State Chinese Medicine Medical Policy Letter [2024] No. 90); Natural Science Research Program for Universities in Anhui Province (2024AH050972).

## Author contributions

R.L. collected the related literature and drafted the manuscript. K.S. supervised the review process. All authors have read and approved the final manuscript.

## CRediT authorship contribution statement

**Kui Sun:** Writing – review & editing, Supervision, Funding acquisition. **Renzhong Li:** Writing – original draft, Validation, Supervision, Investigation, Data curation, Conceptualization.

## Declarations of Interest

The authors declare the following financial interests/personal relationships which may be considered as potential competing interests: Kui Sun reports financial support and article publishing charges were provided by State Administration of Traditional Chinese Medicine. Kui Sun reports financial support was provided by Anhui Provincial Department of Education. If there are other authors, they declare that they have no known competing financial interests or personal relationships that could have appeared to influence the work reported in this paper.

## Data Availability

No data were used for the research described in the article.
